# Clinician Training in the Adaptation of a Comprehensive Tobacco-Free Workplace Program in Agencies Serving the Homeless and Vulnerably Housed

**DOI:** 10.3390/ijerph17176154

**Published:** 2020-08-25

**Authors:** Matthew Taing, Bryce Kyburz, Isabel Martinez Leal, Kathy Le, Tzu-An Chen, Virmarie Correa-Fernandez, Teresa Williams, Daniel P. O’Connor, Ezemenari M. Obasi, Kathleen Casey, Litty Koshy, Lorraine R. Reitzel

**Affiliations:** 1 Department of Psychological, Health & Learning Sciences, The University of Houston, 3657 Cullen Blvd Stephen Power Farish Hall, Houston, TX 77204-5029, USA; mtaing@central.uh.edu (M.T.); imarti31@central.uh.edu (I.M.L.); lek1@livemail.uthscsa.edu (K.L.); tchen3@central.uh.edu (T.-A.C.); vcorreaf@central.uh.edu (V.C.-F.); emobasi@central.uh.edu (E.M.O.); lkoshy@central.uh.edu (L.K.); 2 HEALTH Research Institute, The University of Houston, 4849 Calhoun Rd., Houston, TX 77204, USA; dpoconno@central.uh.edu; 3 Integral Care, Austin, TX, USA; bryce.kyburz@integralcare.org (B.K.); teresa.williams@integralcare.org (T.W.); kathleen.casey@integralcare.org (K.C.); 4 Long School of Medicine, The University of Texas Health Science Center at San Antonio, San Antonio, TX 78229, USA; 5 Department of Health & Human Performance, The University of Houston, 3875 Holman Street, Garrison Gymnasium, Room 104, Houston, TX 77204, USA

**Keywords:** tobacco-free workplace, homeless-serving agencies, tobacco cessation education, tobacco control, homelessness

## Abstract

Tobacco use is exceedingly high among those who are homeless or at risk of homelessness but not commonly addressed by clinicians. Taking Texas Tobacco Free (TTTF) is a tobacco control program that addresses known clinician barriers to intervention (e.g., low training receipt, limited resources). Here, we examine the process and outcomes of TTTF’s adaptation within four agencies that provide housing or other services to individuals who are homeless or vulnerably housed. Pre- and post-implementation data were collected from clinicians (*N* = 68) to assess changes in training receipt, knowledge, and intervention behaviors, relative to program goals. Results indicated significant gains in clinicians’ receipt of training in 9 (of 9) target areas (*p*’s ≤ 0.0042) and a 53% knowledge gain (*p* < 0.0001). From pre- to post-implementation, there were mean increases in the use of the 5As (ask, advise, assess, assist, and arrange) and other evidence-based interventions for tobacco cessation, with significant gains seen in assisting residents/clients to quit, arranging follow-ups, and providing or referring for non-nicotine medications (*p*’s ≤ 0.0491). All program goals, except gains related to advising smokers to quit and the use of specific interventions (behavioral counseling), were met. Overall, TTTF improved clinicians’ capacity to address tobacco use among homeless and vulnerably housed individuals and can serve as a model for tobacco control efforts in similar agencies.

## 1. Introduction

Tobacco use is the leading preventable cause of morbidity and mortality in the United States [1]. Although there have been substantial decreases in the prevalence of cigarette smoking in the last two decades [1], these overall population estimates often fail to account for subpopulations, such as individuals who are homeless or vulnerably housed, that have disproportionately high smoking rates. In fact, over 70% of homeless adults report current smoking—a rate that is approximately five times the national average [2,3]. Moreover, homeless smokers often use dual/multiple (≥2) tobacco products at a rate upward of 68% (compared to 7.9–10.6% among domiciled smokers) [4,5]—a practice that may have more serious implications for health than cigarette-only smoking. When compared to their domiciled counterparts, homeless smokers attempt to quit smoking at comparable rates but have much lower rates of successful quit attempts [6,7], despite a significant portion of this population being interested in quitting [6,8,9,10] and wanting to receive help to do so [8].

Unfortunately, tobacco use is not routinely intervened upon among individuals who are homeless or at risk of homelessness, including in shelters or treatment settings [11]. Consequently, these individuals often have little access to evidence-based pharmacotherapy and behavioral cessation interventions [12,13]. A lack of stable employment and health insurance [14] further precludes the receipt of evidence-based care. There are also known barriers reported by clinicians that affect the provision of care, including a lack of knowledge about how to treat tobacco use behaviorally or pharmacologically, inadequate time to address tobacco use, and/or a lack of available resources to distribute to residents/clients [15,16,17,18,19]. The result of this unaddressed disparity is high rates of premature disability and death related to tobacco use [20,21,22]. Evidence-based tobacco control initiatives are thus critical to reducing tobacco use and tobacco-related morbidities—such as cancers, heart disease, stroke, and chronic obstructive lung diseases [23]—among this vulnerable and underserved population.

Tobacco-free workplace (TFW) programs are evidence-based, tobacco control initiatives that may be effective in increasing the capacity to address tobacco use within homeless-serving community agencies. TFW programs protect employees, residents/clients, and visitors from environmental tobacco smoke exposure, while also encouraging current and former smokers to maintain smoking abstinence through no tobacco use on site policies [24,25,26,27]. Effective implementation of tobacco control initiatives includes (1) stakeholder education about the harms of tobacco use and how to address it within the context of their organization [28]; (2) organizational-level policies that foster and enforce tobacco-free environments [29]; and (3) specialized training for clinicians (e.g., behavioral counseling) [30]. However, homeless-serving agencies may be reluctant to enact certain evidence-based components of TFW programs, such as prohibiting smoking/vaping on site. This is because policy enforcement might mean stakeholder eviction or fines that could potentially affect individuals’ use of residential or other services. Other feared consequences can include residents/clients wandering off site to use tobacco and possibly increasing the potential for negative interactions with the community and/or police, and/or engendering complaints from surrounding residences and businesses about cigarette trash (e.g., butts). Thus, agencies serving individuals and families who are homeless or vulnerably housed may not consider the implementation of TFW programs as feasible within their settings, despite the fact that tobacco control interventions are sorely needed for these groups and that other elements of a TFW program would not run counter to the agencies’ values.

Taking Texas Tobacco Free (TTTF) is an academic–community partnership between faculty at the University of Houston and employees of Integral Care, a local mental health authority (LMHA) in Austin. TTTF has been funded by the Cancer Prevention and Research Institute of Texas to assist partnering agencies to implement a multi-component TFW program by (1) educating clinicians and staff about the importance of a TFW policy and about the hazards of tobacco use; (2) facilitating TFW policy development, implementation, and enforcement; (3) offering specialized training for clinicians on regularly screening for tobacco use and using evidence-based practice delivery to address tobacco use; and (4) providing resources to facilitate successful implementation and cessation (e.g., nicotine replacement therapy (NRT), permanent workplace signage, and passive dissemination materials). As such, TTTF represents a model for a comprehensive, multi-component TFW approach that is able to have larger impacts on population health than mono-component strategies [29]. Although complete TFW policies are more impactful than partial policies (e.g., prohibiting tobacco use indoors only, limiting tobacco use to designated areas), TTTF uses an agency-specific implementation approach. Thus, an assessment of an agency’s assets and barriers is undertaken to facilitate a toolkit model whereby the program can be implemented within agencies with varying levels of tobacco control programming (e.g., some or no programming in place) or agencies seeking to improve the quality, scope, or implementation of existing tobacco control policies and practices.

To date, TTTF has been successfully implemented in 23 LMHAs, comprising approximately 300 mental health centers [27,31,32,33,34,35]. TTTF received additional funding in 2017 to expand into standalone substance use treatment centers and other community agencies, including those providing services and care to individuals who are homeless or vulnerably housed. This process required the adaptation of the TTTF program to address a more diverse clientele across participating centers. Our work on the latest grant is ongoing, but initial adaptations for substance use treatment centers have demonstrated success relative to goals [35], as well as the importance of clinician training in building agency capacity to address tobacco use. In scaling up the intervention to be more appropriate for settings serving individuals who are homeless and vulnerably housed, TTTF implementation focused on known clinician barriers to cessation intervention provision given the commonality of lack of information and resources reported among clinicians in settings where tobacco use rates among clientele are high [15,16,17,18,19]. Thus, we had the following goals: (1) increase tobacco education training receipt among clinicians; (2) achieve increases in knowledge gained from tobacco education among clinicians; (3) provide specialized training to clinicians on treating tobacco use; (4) increase clinicians’ self-reported use of the 5As (ask, advise, assess, assist, and arrange) [30] and the provision of empirically based interventions for tobacco users; and (5) distribute population-tailored, resident-/client-focused passive dissemination materials with information on the consequences of tobacco use and how to get help with cessation (e.g., quitlines). Further, in healthcare service agency settings where treatment was provided on site, TTTF had additional goals to (6) increase capacity for clinician’s provision of tobacco use assessments (TUAs); and (7) increase capacity for clinician’s delivery of evidence-based tobacco cessation pharmacological interventions (i.e., NRT). Benchmarks for achieving these goals were set based on a literature review [36,37,38] and our prior implementation work [27,31,32,33,34,35] and were approved by our funder. To our knowledge, no prior publications have focused on adaptations of TFW programs for application within agencies serving individuals who are homeless and vulnerably housed.

The purpose of the current study is to examine the process and outcomes of the TTTF program’s adaptation for agencies serving individuals who are homeless or vulnerably housed. Herein, we describe our implementation of a TFW program in these settings, with a specific focus on clinician training, and the results of our efforts relative to our stated goals. We expected that it would be feasible to implement TTTF in these settings and anticipated it would provide a working model for future implementation in similar agencies.

## 2. Materials and Methods

### 2.1. Participating Agencies

The leadership of four agencies providing services to individuals who were homeless or vulnerably housed were approached about their interest in participating in the TTTF program. Agencies were selected due to prominence in serving the homeless or vulnerably housed in cities where the study team was physically located (Houston and Austin, TX, USA). The four participating agencies were a subset of community agencies serving a variety of vulnerable populations who participated in the program; however, these four participating agencies were the only ones enrolled that served homeless or vulnerably housed individuals. Agency 1 provides services to homeless individuals and families in the areas of education, employment, and housing in Houston. Agency 2 provides affordable housing and support services for families that have very limited resources to prevent homelessness in Houston. Agency 3 provides accessible and comprehensive healthcare and other services to homeless individuals in Houston. Agency 4 provides affordable housing and support services for a variety of individuals who are homeless or vulnerably housed in Austin. In each case, residence at the agencies was not required for the receipt of services. Thus, individuals considered “homeless” for the purpose of this paper comprise those living in shelters as well as those sleeping “rough” (unsheltered, on the streets), the majority of whom meet the U.S. Department of Housing and Urban Development definition of “literally homeless” and “homeless under other federal statutes” [39]. Individuals considered “vulnerably housed” for the purposes of this study are those “at imminent risk of homelessness” [39] and those who are living within an income-restricted supportive housing property for vulnerable families with children or within single-room occupancy residency units.

### 2.2. Participating Clinicians and Their Reach

Participating agencies reported 63 client-facing staff and 454 non-client-facing (general) employees at implementation. Our adaptation of TTTF focused on educating/training client-facing staff based on their ability to provide interventions for tobacco use with their residents/clients. This group (hereafter referred to as “clinicians”) included individuals from various professional and educational backgrounds whose job duties included engaging and intervening with agency residents/clients on matters of health and wellness. Some clinicians provided treatment full time within the context of offices within the agency itself, whereas others were primarily focused on providing case management or outreach services to clients on the streets. Together, the agencies served 23,405 unique residents/clients through 93,208 annual contacts, as per their recent annual reports (see Table 1).

### 2.3. Procedures

Study procedures were approved by the IRB at the University of Houston (STUDY00000472, approval date 7/27/17). Leaders of participating agencies signed a memorandum of understanding delineating their agreement to participate in the program. Thereafter, a modified TTTF program was implemented in each setting over a 12–15 month period (mean ± SD = 14.31 ± 1.56 months). First, 1.5–2 h trainings on tobacco control and tobacco cessation, tailored to focus on research and issues relevant to individuals who were homeless or vulnerably housed, were presented to all clinicians. These trainings were held on site. TTTF also sponsored a small group of clinicians with representation from each agency to attend a 4-day, expenses paid, Certified Tobacco Treatment Specialist Training in order to embed specialized knowledge for delivering TUAs and treating tobacco use in the agency itself. A 7.5 h Motivational Interviewing training [40] was offered to each participating agency for clinicians to learn how to motivate quit attempts in their work and outreach. Agency 3 declined this training because it was already offered in-house. Each agency received passive dissemination materials (e.g., posters and rack cards) tailored for homeless and vulnerably housed individuals to display in their facilities and distribute to residents/clients. Agency 3 was the only agency with direct treatment capacity and as such took advantage of NRT from TTTF. Agency 3 also received a carbon monoxide monitor and supplies, with training provided on how to use it as a tool to motivate quit attempts. While prior TTTF implementations also included the initiation of a completely TFW policy and accompanying signage [27,31,32,33,34], agency leadership expressed an unwillingness to participate in TTTF if this were mandated. Their concerns were based on their values and mission as the last resort for many individuals and families, from which they did not want to exclude active tobacco-users or levy penalties against them (e.g., fines, eviction) for policy violations. Thus, TTTF waived this requirement for the participating agencies in order to facilitate their engagement.

### 2.4. Measures

#### 2.4.1. Tobacco Education Training Receipt (Goal 1)

An online pre-implementation survey was distributed to clinicians at each agency by the program “champion” (a designated employee who was the agency liaison with the TTTF program) prior to implementing any aspects of the TTTF program. Likewise, an online post-implementation survey was administered after all implementation procedures were enacted. These investigator-generated, face-valid surveys queried training receipt in various topics, reflective of TTTF programming content and training goals, which were based on empirically supported recommendations for comprehensive tobacco-free workplace program implementations [36,37,38]. Items on these surveys included: “In the last 12 months, have you received any training on the use of counseling and behavior therapies to treat tobacco use?” with response options of yes or no. The remainder of these items are in Table 2. TTTF staff would monitor the receipt of completed electronic surveys and send reminders to the program champion to request they encourage greater participation, as needed.

Our explicit goal was to achieve endorsement that ≥60% of responding clinicians at the participating agencies had received specialized training in tobacco dependence in the post-implementation survey. Although we did not know baseline rates of training receipt when we set our grant goals, we estimated from the literature that ≤30% of clinicians would report such training in the months prior to program implementation.

#### 2.4.2. Knowledge Gain (Goal 2)

A 10-item, investigator-generated knowledge test was administered before and after the 1.5–2 h clinician training to measure knowledge gained from the training. Items included “What strength of nicotine patch should be used for a person who is smoking a pack of cigarettes per day?” (response options = 28, 21, 14, and 7 mg) and “Tobacco-free campus policies will lead to premature withdrawal from programs at significant levels” (response options = true or false). Items were face-valid and directly reflected educational content that was explicitly reviewed in the training. In each case, these paper-based test items were distributed by TTTF staff immediately prior to the training session and immediately following the training sessions. Respondents endorsed their responses by filling in the corresponding letter on a bubble sheet that was labeled “pre” or “post” to facilitate the staff’s organization of the sheets for analysis. No personal identifying information was requested on these bubble sheets; thus, pre- and post-test/survey comparisons were not matched at the level of the respondent but instead were collated by agency.

Our explicit goal was to achieve a 50% increase in attendee knowledge from pre-training to post-training.

#### 2.4.3. Specialized Training Provision (Goal 3)

Receipt of specialized Certified Tobacco Treatment Specialist Training and Motivational Interviewing Training was each assessed based on TTTF program records (sign-in sheets, attendance verified by trainers).

Our explicit goal was to embed specialized knowledge within each participating agency by having at least 1 employee fully attend the 4-day Certified Tobacco Treatment Specialist Training and by having ≥60% of clinicians at each agency attend the 1-day Motivational Interviewing training.

#### 2.4.4. Clinician Intervention Provision (Goal 4)

The same pre-implementation and post-implementation surveys relevant to Goal 1 also included items querying clinicians’ self-reported use of the 5As [30] and the provision of empirically based interventions for tobacco users. Items included “With regard to clientele that you saw last month who smoked, did you advise them to quit smoking?” with response options of yes or no. The remainder of these items are in Table 3.

There was no specific goal set for participating agencies in this area of adoption, as in-office treatment with chart documentation was not ubiquitous; however, a goal we have used within our prior implementations with LMHAs was to achieve ≥65% of responding clinicians reporting the delivery of evidence-based tobacco dependence intervention to consumers at post-implementation, which could be generalized here, as applicable.

#### 2.4.5. Passive Dissemination (Goal 5)

The TTTF team created three poster designs featuring images of individuals who were homeless with messages about quitting tobacco use and directions regarding how to quit, as informed by suggestions from partnering agencies. Specifically, the qualitative scientist on the TTTF team consulted with employees at the participating agencies and showed them materials we had developed to date for implementation in LMHAs. Agency employees suggested we find more “real” images showing individuals who were “down and out, homeless-looking people, not happy, smiling people who looked like they’d never seen a day of trouble in their lives.” Based on this information, the team selected and purchased images fitting the request, and materials were then sent to agency leadership for additional suggestions, which led to the selection of three final acceptable, representative images for the design. Each poster included the number to the state’s tobacco quitline for more help. Dissemination materials were to be posted in the agencies’ areas where residents/clients would see them (e.g., each treatment room, around the front desk). The purpose of these materials was to encourage residents/clients to quit tobacco use, demonstrate agency support for a tobacco-free culture and to remind employees where they could direct residents/clients for help. TTTF personnel queried agency capacity for passive dissemination materials and delivered them to the agencies for posting.

There was no specific program goal regarding how many materials would be disseminated, but we aimed to provide 100% of materials requested by the program partners as part of the implementation.

#### 2.4.6. Tobacco Use Assessments and Nicotine Replacement Therapies (Goals 6 and 7)

Following implementation, Agency 3 (the healthcare service agency) completed four quarterly reports of the number of TUAs delivered and NRTs distributed to residents/clients, which represented activities over the year following TTTF implementation procedures. Additionally, perceived effectiveness with regard to delivering TUAs was assessed at post-implementation only through an item on the aforementioned survey (see Goals 1 and 4) querying degree of agreement to a statement about being “able to effectively deliver TUAs to clientele” using a five-point Likert scale.

Our specific goals were that ≥80% of agencies with capacity to conduct TUAs were compliant with reporting them on a quarterly basis, and that NRT would remain available to clientele at least 6 months following program implementation. Although there was no specific goal regarding reported effectiveness in delivering TUAs to clientele, the project team aimed that the majority of respondents would endorse perceived effectiveness at post-implementation.

### 2.5. Analytic Plan

Many program goals were assessed with simple proportions of responses at post-implementation that were achievable using division. However, we additionally wished to examine the statistical significance of changes in our metrics over time. Thus, to examine pre- to post-implementation differences in training receipt (Goal 1) and clinician intervention provision (Goal 4), Cochran–Mantel–Haenszel tests were used, controlling for the implementation site (i.e., agency). Knowledge gain from the 1.5–2 h clinician training was assessed by scoring pre- and post-knowledge tests as far as number of items correct (possible range 0–10) on average. An analysis of covariance, controlling for agency, was used to evaluate the statistical significance of knowledge gains from pre- to post-training (Goal 2). Additionally, as indicated by program goals, the percent increase in knowledge (increase ÷ original × 100) across agencies was also calculated. TTTF personnel tallied the number of attendees to specialized trainings (Goal 3) and the number of passive dissemination materials given to the agencies (Goal 5), respectively, across the implementation period. Quarterly report data on TUA administration and NRT distribution (Goals 6 and 7) were also tallied. All statistical analyses were conducted using SAS version 9.4 [41], with alpha set at 0.05.

## 3. Results

The first program goal was that ≥60% of responding clinicians reported the receipt of specialized training at post-implementation, which we estimated, based on the literature, would represent an increase in training receipt from pre-implementation. Overall, 53 clinicians (~84% of the originally reported 63) completed the pre-implementation survey and 27 clinicians (~42.9%) completed the post-implementation survey. In both cases, not all questions were answered by all respondents. From pre- to post-implementation, clinicians reported significantly greater exposure to recent training in the topic areas assessed as indicated in Table 2. Moreover, the goal of achieving ≥60% of respondents indicating the receipt of this training post-implementation was achieved.

The second program goal was to achieve increases in knowledge gained from tobacco education, as measured by a 10-item knowledge test. Overall, 68 clinicians across the agencies took the pre-training knowledge test and 65 took the post-training test. This accounts for >100% of the reported number of client-facing staff (*N* = 63), which may be accounted for by new hires since pre-implementation or a few hand-selected general/supervisory employees who also wanted to attend. The average pre-training knowledge test score was 4.97 ± 1.88 and the average post-training knowledge test score was 7.62 ± 2.39. Overall, results indicated a 53% knowledge gain from pre-training to post-training, representing a statistically significant change (*p* < 0.0001). Thus, our program goal of ≥50% knowledge gain was achieved.

The third program goal was to provide specialized training to clinicians on treating tobacco use. Overall, 1–2 clinicians from each agency (*N* = 5 total) fully attended the Certified Tobacco Treatment Specialist Training and 41 clinicians (41/63 = 68.3%) attended the Motivational Interviewing training. Thus, the program goal was achieved for both the Certified Tobacco Treatment Specialist Training and the Motivational Interview training.

The fourth program goal was to increase clinicians’ self-reported use of the 5As and the provision of empirically based interventions for smoking residents/clients. From pre- to post-implementation, clinicians reported mean increases in their use of the 5As and significantly greater efforts to assist users to quit (*p* = 0.0316) and arrange follow-up about the quit attempts (*p* = 0.0175). Likewise, mean increases were seen in the provision of empirically supported treatments to smokers with the only significant change arising in the use of non-nicotine-based medications or referral for such (*p* = 0.0491) (see Table 3). The goal here was to achieve the delivery of these empirically supported interventions by ≥65% of responding clinicians at post-implementation, which was not achieved for “advising smokers to quit” or for the provision of specific interventions, such as “behavioral counseling.”

The fifth program goal was to distribute passive dissemination materials to participating agencies. Overall, 4131 tailored materials encouraging residents/clients to quit tobacco were distributed to the agencies. This represented the number of materials requested from the agencies, thus achieving our dissemination goal. To contextualize this, it represents one poster displayed at the settings for every 17 (4131/23,405 = 17.7) residents/clients.

Finally, the sixth and seventh program goals were to increase agency capacity for TUA administrations and NRT distribution, respectively. The single participating agency with direct treatment capacity did not comprehensively screen or follow-up on tobacco use in clinical encounters or routinely offer NRT to tobacco-using clients prior to program participation. This agency was given a “starter kit” of ~$22,500 worth of NRT (gum and patches), distributed in three shipments across a 5 months span for its clients. Quarterly report data indicated that this agency screened 3195 unique individuals for tobacco use via a TUA (Q1 = 95; Q2 = 1699; Q3 = 1481; Q5 = 1419) and provided NRT to 265 individuals (Q1 = 0; Q2 = 103; Q3 = 102; Q4 = 60) who wanted to quit during the year following implementation. Overall, 23.08% (*N* = 6) of clinicians “strongly agreed” and 57.69% (*N* = 15) of clinicians “agreed” at post-implementation that they were “able to effectively deliver TUAs to clientele,” with 11.54% (*N* = 3) neither agreeing nor disagreeing, 7.69% (*N* = 2) disagreeing, and 0% strongly disagreeing. Thus, program goals were achieved whereby the single agency with the capacity for such (i.e., 100% of applicable agencies) was compliant with quarterly TUA reporting and NRT distribution reporting following implementation. Moreover, the majority of clinicians reported the ability to effectively deliver TUAs to clientele, as desired.

## 4. Discussion

This study described the process and outcomes of the adaptation of TTTF, a comprehensive TFW program, in four agencies serving individuals who were experiencing homelessness or at risk of homelessness within two large metropolitan cities in Texas. Results support pre- to post-implementation increases in receipt of tobacco education training among clinicians with statistically significant knowledge gains, increases in clinicians’ self-reported use of the 5As (ask, advise, assess, assist, and arrange) [30], increases in the provision of empirically based interventions for tobacco-using residents/clients; and increased capacity for clinicians’ provision of TUAs and NRT (the latter at the single agency that provided such services). Moreover, TTTF provided specialized training to clinicians on treating tobacco use and distributed population-tailored, resident/client-focused passive dissemination materials with information on the consequences of tobacco use and how to get help with cessation (e.g., quitlines). Thus, results support the feasibility of the implementation of TTTF in agencies serving the homeless and vulnerably housed and provide a model for a TFW program implementation that addresses known clinician barriers to addressing tobacco use [15,16,17,18,19]. Consequently, and knowing that TFW program implementations are ultimately effective in reducing tobacco use among setting stakeholders [31,32,36,42], authors suggest that programs such as TTTF may provide opportunities for reducing tobacco use disparities among vulnerable groups [24,25,26,27,30,35]. However, more research is needed to determine whether such programs are effective in reducing tobacco use among populations specifically served by agencies such as the ones described here. Additionally, as this study was limited to Texas agencies, results may not be generalizable to community agencies in other states. Thus, program goals may require modification to meet local requirements and needs. In addition, it is important to note areas for improvement in program implementation. For example, participation in evaluation procedures was not universal, response rates—particularly at post-implementation—were not ideal, not all pre- to post-implementation changes were statistically significant, TUAs/interventions were conducted by fewer than 100% of the targeted clinician stakeholders, and client-level data regarding changes in tobacco use were not collected to confirm presumed effects on cessation [31,32,36,42]. Although these limitations are not uncommon in similar, real-world implementation/training efforts (e.g., [15,43]), they provide areas that can be strengthened in future implementation (or bolster) efforts.

From pre- to post-implementation, participating clinicians reported significantly greater exposure to tobacco-related training and experienced increases in knowledge on treating tobacco use. This was expected and is congruent with previous research finding that providing general education trainings can lead to short-term increases in provider knowledge and more positive attitudes toward smoke-free policies [15,33,44]. Although clinicians generally receive basic tobacco cessation training in their formal education [18], having basic knowledge may not translate into effective cessation clinical skills [18]. Thus, continuing education as a professional may be necessary to reinforce existing knowledge and to encourage familiarity with changes in literature related to tobacco use cessation and emerging tobacco products and treatments. In the current study, the vast majority of responding clinicians (~80%) indicated agreement that they were “able to effectively deliver TUAs to clientele” at post-implementation. By increasing self-efficacy through education, clinicians are more likely to feel confident in their ability to treat clients with evidence-based practices and/or to motivate quit attempts [16,19,43], and our findings largely suggest clinician self-efficacy in TUA delivery (though we lacked a pre-implementation measure of efficacy to assess change over time). A limitation of the current work, however, is that measures of knowledge gain were taken immediately before and after training; thus, the trainings’ effects on knowledge retention over time is unknown. Future studies should consider collecting follow-up data among clinicians and potentially seek to reinforce identified gaps in knowledge through bolster trainings over time or the incorporation of tobacco intervention training in annual and new employee trainings. The TTTF program currently provides agencies with continuing education resources through our website [34]; however, at least some work among behavioral health treatment providers suggests that in-person or face-to-face trainings are preferred [19]. Moreover, the integration of continuing education into the agency’s regular procedures (e.g., during new employee orientation, etc.) may help to address knowledge loss as the results of clinician turnover experienced during or following the active implementation. Such programs could be delivered internally by the employee who completed the Certified Tobacco Treatment Specialist training, for example. In addition to gathering longer-term follow-up data on clinicians’ knowledge and use of empirically supported interventions for tobacco cessation, linked information on clinician characteristics (e.g., their own smoking status) would also be interesting to explore as moderators of learning and behavioral outcomes. Additionally, future studies should seek to collect client-level data related to demographic characteristics, tobacco use, quit attempts, and abstinence duration. However, real-world challenges to the collection of these data will need to be considered, including that some agencies or clinicians may not participate if requests for information become time consuming and the potentially transient nature of the target population (individuals who are homeless). The provision of financial incentives for participation may help to mitigate these challenges to some degree [45,46,47].

Trainings to treat tobacco dependence in agencies serving individuals who are vulnerably housed or experiencing homelessness are essential to changing treatment culture in these settings [38]. Tobacco use screening and interventions remain among the top preventative services that can be offered to adults in regard to cost effectiveness and preventable burden of disease [18,48]. The basis for many tobacco cessation services is the use of the 5As [38]. In the current study, rates of clinician compliance with the administration of each of the 5As post-implementation with tobacco-using residents/clients ranged between 63% and 79%, leaving room for improvement, especially in the area of “advising smokers to quit,” which did not meet the delivery goal for ≥65% of clinicians. Previous studies have found that implementation of the full 5As is time consuming [43,49], and have recommended a 2As and R model (AAR: ask/advise/refer) [38,43]. Future work in this area might examine whether the AAR alternative may be helpful to improve compliance, particularly in agencies without on-site cessation services. Moreover, despite the length of time between our pre- and post-implementation assessments (mean ± SD = 14.31 ± 1.56 months), future work would benefit from later post-implementation follow-ups to assess for any decreases in assessment and treatment provision. Nevertheless, results indicated mean increases in each of the 5As, with significant increases in assisting clientele to quit and arranging follow-ups. Despite the lack of statistically significant gains in some areas, changing clinician behaviors around addressing tobacco use with this vulnerable group of resident/client stakeholders may certainly be clinically significant.

Prior work indicated that difficulty making time for training and a lack of access to tobacco cessation dissemination materials resources were barriers to the screening and treatment of tobacco use in behavioral health settings [19]. Despite time constraints, the TTTF program was able to engage clinicians at the targeted agencies to attend trainings, some of which required 1 day (Motivational Interviewing) or 4 days (Certified Tobacco Treatment Specialist training) out of the office and away from residents/clients. The buy-in from agency leadership through the memorandum of understanding regarding program requirements was likely quite helpful in enabling this and seems an important aspect of future implementation. Likewise, TTTF’s distribution of tailored, passive dissemination materials to agencies addressed a known barrier to intervention [19] and may have also facilitated referrals by providing easy-access information to residents/clients regarding the dangers of smoking, smoking among various vulnerable populations, and resources to help facilitate quit attempts. Research suggests that when given sufficient information about the dangers of tobacco use, clients are more inclined to reflect on their own behaviors and potentially seek help [50]. Thus, the TTTF program made dissemination materials available online for ease of download following the active implementation period [34], wherein printed materials were delivered directly to the agencies. Implementation in other locales/agencies can make use of this downloadable material or request brochures from other advocacy organizations who might freely provide print materials.

One area for improvement in this TTTF implementation is with regard to the provision of treatment to tobacco-using stakeholders. Even at post-implementation, clinician compliance with treatment provision to tobacco-using stakeholders ranged from 29% for non-nicotine based medications or referral for medications to 48% for the provision of behavioral counseling; these specific interventions fall short of our goal for delivery among ≥65% of clinicians and an overall ideal of 100% participation. To some degree, this may have been reflective of an unwillingness to quit tobacco use during a specific clinical encounter, which is not uncommon given motivation to quit may wax and wane over time and potentially even within a given day [51]. Thus, highlighting the need to continually engage tobacco-using residents/clients about interest in quitting is an important aspect of clinician training, as well as developing clinician skills in motivating a quit attempt. Non-compliance with evidence-based medication provision as seen in our results may also reflect the nature of the agency (primary mission to house versus treat) or the clinicians’ job responsibilities (outreach to provide critical services such as access to food versus treatment provision). Future work in this area might benefit from qualitative work pre- (and post-) implementation to ascertain agency specific barriers to empirically based treatment provision that might be addressed during the implementation process to enhance clinician compliance with treatment provision.

Best practices for tobacco control encompass both clinical (e.g., cessation services) and public health (e.g., policies) strategies to encourage cessation and discourage use of tobacco products [38,52]. TTTF’s implementation resulted in direct changes in clinical strategies with self-reported increases in the use of the 5As and evidence-based practices (e.g., behavioral counseling, NRT or referral for NRT, and non-nicotine-based medication or referral for such). When compared to TTTF’s prior implementation in LMHAs, there were similar increases in the use of components of the 5As and delivery of evidence-based practices (specifically, provision or recommendation for NRT) [32]. These similarities further support the feasibility of TTTF’s tailored implementation in agencies serving vulnerable groups. Prior to TTTF’s implementation, the single agency with direct treatment capacity did not regularly provide comprehensive TUAs or distribute NRTs. Following implementation, this agency was able to use and expand upon existing questions in their electronic health record system to consistently assess tobacco use and assist quit attempts among clients with the desire to quit. Thus, TTTF’s implementation helped to establish processes that emphasized the importance of TUAs and enhanced agency capacity to address tobacco use consistently among stakeholders. With regard to policy, however, TTTF was not able to implement its typical 100% tobacco-free workplace in any of the participating agencies, wherein designated smoking areas on the agency campus were disallowed/removed. Agency leadership indicated reluctance to implement such policies because they typically carry penalties for failure to comply, including fines and possibly eviction (as applicable) for routine violations. Given these agencies’ nature as a “lifeline” for care and resources to vulnerable groups, imposing such penalties as part of policy enforcement was perceived as contradictory to their values and mission. As such, it may be best to consider harm reduction strategies, wherein policies such as partial smoking bans or voluntary adoption (by residents) are implemented. Previous studies that looked at the implementation of a partial smoking ban in a shelter setting [53] and voluntary smoke-free policy adoption in permanent supportive housing [54] have demonstrated moderate success. Such strategies may not motivate substantial quit attempts [53], but may represent first steps to increase resident/client (and agency) buy-in toward becoming 100% tobacco free and ultimately changing the smoking culture in these settings. Other options may include brainstorming alternate methods of policy enforcement with willing agencies that are perceived as more palatable to stakeholders and/or explore methods of positive reinforcement for policy compliance that may help to extinguish tobacco use on site. In any case, we recommend that the enforcement of a tobacco-free policy is coupled with the provision of empirically supported resources for quitting to support residents/clients and that policy changes are transparent, announced in advance, and accompanied by efforts to engage and educate those who will be affected (e.g., via stakeholder town hall meetings) [27].

## 5. Conclusions

In summary, the TFW program model provided here serves as a framework for similar community agencies to adapt evidence-based tobacco control programs and to expand sustainable initiatives aimed at reducing tobacco use. Increases in clinician exposure to training, knowledge, and use of evidence-based practices indicate TTTF’s feasibility and successful implementation in agencies serving the homeless and vulnerably housed. Future work should examine the generalizability of results within other settings/locales, acceptable methods of limiting tobacco use on agency grounds through policy enactment, effects of the program on residents’/clients’ quit rates, the cost-effectiveness of this work, and the usefulness of bolster trainings or other methods to address clinician turnover and ensure the longevity of clinician behavioral changes in addressing tobacco use among underserved and vulnerable groups.

## Figures and Tables

**Table 1:** Employees and annual client contacts in enrolled agencies.

Enrolled Agency	Clinicians	General Employees	Unique Clients Served	Annual Contacts
Agency 1	15	110	4326	35,452
Agency 2	12	58	1200	1200
Agency 3	20	68	4500	35,000
Agency 4	16	218	13,379	21,556

**Table 2:** Training receipt from pre- to post-Taking Texas Tobacco Free (TTTF) implementation among responding clinicians.

Training Query Items	Pre	Post	*p*-Value
In the Last 12 Months, Have You Received Any Training On…	% (*n*) Endorsing Yes *		
Assessing clients for their tobacco use ^╪^ (Pre: 96.2%, Post: 88.9%)	23.5 (12)	75.0 (18)	<0.0001
Treating tobacco use in conjunction with SUDs ^╪^ (Pre: 96.2%, Post: 88.9%)	23.5 (12)	62.5 (15)	0.0022
How quitting tobacco improves substance use recovery ^╪^ (Pre: 96.2%, Post: 92.6%)	27.4 (14)	76.0 (19)	0.0002
How continued substance use may be a barrier to quitting tobacco ^╪^ (Pre: 92.5%, Post: 92.6%)	24.5 (12)	72.0 (18)	0.0002
The use of pharmacotherapies (e.g., NRT, Chantix) to treat tobacco use ^╪^ (Pre: 96.2%, Post: 92.6%)	17.6 (9)	80.0 (20)	<0.0001
The effects of tobacco smoke on psychiatric medications ^╪^ (Pre: 96.2%, Post: 92.6%)	17.6 (9)	52.0 (13)	0.0042
How tobacco may be used to cope with the side effects of psychiatric meds ^╪^ (Pre: 96.2%, Post: 92.6%)	17.6 (9)	68.0 (17)	<0.0001
The hazards of smoking and benefits of quitting for individuals with SUDs ^╪^ (Pre: 96.2%, Post: 92.6%)	19.6 (10)	72.0 (18)	<0.0001
The use of counseling and behavior therapies to treat tobacco use (e.g., MI) ^╪^ (Pre: 96.2%, Post: 92.6%)	39.2 (20)	84.0 (21)	0.0004

Note: * Respondents could skip items not relevant to their job duties; for this reason, percentages are calculated based on the number of item respondents; SUDs: substance use disorders; NRT: nicotine replacement therapies; MI: motivational interviewing; ^╪^ response rate.

**Table 3:** Intervention provision from pre- to post-TTTF implementation among responding clinicians.

Intervention Query Items	Pre	Post	*p*-Value
**With regard to clientele that you saw last month who smoked, did you…**	% (*n*) endorsing yes *		
Ask clientele about their smoking status? ^╪^ (Pre: 100%, Post: 96.3%)	49.1 (26)	69.2 (18)	0.2210
Advise them to quit smoking? ^╪^ (Pre: 86.8%, Post: 81.5%)	54.3 (25)	63.6 (14)	0.3172
Assess their willingness to make a quit attempt? ^╪^ (Pre: 86.8%, Post: 88.9%)	67.4 (31)	79.2 (19)	0.5403
Assist them to quit by providing treatment or making a referral for treatment? ^╪^ (Pre: 86.8%, Post: 85.2%)	43.5 (20)	73.9 (17)	0.0316
Arrange to follow-up with them to assess progress regarding smoking cessation? ^╪^ (Pre: 86.8%, Post: 85.2%)	39.1 (18)	73.9 (17)	0.0175
**What types of treatment do you typically provide for smokers or other tobacco users?**	% (n) endorsing yes *		
Behavioral counseling	43.4 (23)	48.1 (13)	0.9345
NRT (e.g., patch, gum) or referral for such	17.0 (9)	40.7 (11)	0.0512
Non-nicotine-based medications (e.g., Chantix) or referral for such	9.4 (5)	29.6 (8)	0.0491
I do not typically provide treatment for smokers or other users	52.8 (28)	40.7 (11)	0.5436

Note: * Respondents could skip items not relevant to their job duties; for this reason, percentages are calculated based on the number of item respondents; NRT: nicotine replacement therapies; ^╪^ response rate.
